# Religious and spiritual interventions in mental health care: a systematic review and meta-analysis of randomized controlled clinical trials

**DOI:** 10.1017/S0033291715001166

**Published:** 2015-07-23

**Authors:** J. P. B. Gonçalves, G. Lucchetti, P. R. Menezes, H. Vallada

**Affiliations:** 1 Department of Psychiatry, University of Sao Paulo Medical School, Sao Paulo, SP, Brazil; 2 Federal University of Juiz de Fora, Juiz de Fora, Minas Gerais, Brazil; 3 Department of Preventive Medicine, University of Sao Paulo Medical School, Sao Paulo, SP, Brazil

**Keywords:** Clinical care, clinical trials, meta-analyses, religiosity, spirituality

## Abstract

**Background.:**

Despite the extensive literature assessing associations between religiosity/spirituality and health, few studies have investigated the clinical applicability of this evidence. The purpose of this paper was to assess the impact of religious/spiritual interventions (RSI) through randomized clinical trials (RCTs).

**Method.:**

A systematic review was performed in the following databases: PubMed, Scopus, Web of Science, PsycINFO, Cochrane Collaboration, Embase and SciELO. Through the use of a Boolean expression, articles were included if they: (i) investigated mental health outcomes; (ii) had a design consistent with RCTs. We excluded protocols involving intercessory prayer or distance healing. The study was conducted in two phases by reading: (1) title and abstracts; (2) full papers and assessing their methodological quality. Then, a meta-analysis was carried out.

**Results.:**

Through this method, 4751 papers were obtained, of which 23 remained included. The meta-analysis showed significant effects of RSI on anxiety general symptoms (*p* < 0.001) and in subgroups: meditation (*p* < 0.001); psychotherapy (*p* = 0.02); 1 month of follow-up (*p* < 0.001); and comparison groups with interventions (*p* < 0.001). Two significant differences were found in depressive symptoms: between 1 and 6 months and comparison groups with interventions (*p* = 0.05). In general, studies have shown that RSI decreased stress, alcoholism and depression.

**Conclusions.:**

RCTs on RSI showed additional benefits including reduction of clinical symptoms (mainly anxiety). The diversity of protocols and outcomes associated with a lack of standardization of interventions point to the need for further studies evaluating the use of religiosity/spirituality as a complementary treatment in health care.

## Introduction

Despite the interconnection throughout history between religion, spirituality and medical practice, only in the last decades has the scientific literature demonstrated the important role of religiosity/spirituality (R/S) in the physical and mental health of patients (Koenig *et al.* 2012).

However, defining complex and multifaceted concepts such as spirituality and religiosity is not easy as there is no universal definition accepted by researchers (Cook, 2004). Sullivan (1993) defined spirituality as an individual and unique feature that links the self to the universe and to others, and may or may not include a belief in a god. Puchalski (2012) describes spirituality as a way to find meaning and purpose in life by connecting the inside with the sacred. In addition, Koenig *et al.* (2012) define spirituality as ‘distinguished from humanism, values, morals, and mental health, by its connection to which is sacred, the *transcendent*’ and that religion ‘involves beliefs, practices, and rituals related to the *transcendent*, where the transcendent is God’.

This lack of consensus causes difficulty in comparing the results between studies (Lucchetti *et al.* 2013). Nevertheless, several studies have shown positive correlations between R/S and the prevention of various diseases with evidence of improved quality of life and increased survival (Sawatzky *et al.* 2005; Chida *et al.* 2009).

Different papers have reported a correlation between greater religious attendance and increased immunity (Bormann & Carrico, 2009), lower blood pressure and cardiac complications in postoperative patients (Lucchetti *et al.* 2011; Masters & Hooker, 2013) and correlation with remission of cancer (Ando *et al.* 2010; Ka'opua *et al.* 2011).

Regarding mental health, some studies have shown a direct relationship with psychological well-being, such as satisfaction, happiness and moral values (Bonelli *et al.* 2012; Moreira-Almeida *et al.* 2014). Koenig *et al.* (2012) in their review reported a 95% positive correlation with social support, 93.7% with purpose and meaning of life and 79% with well-being, optimism and hope.

However, despite numerous positive correlations, there are also reports of negative aspects of religiosity that are associated with thoughts of guilt, abandonment or punishment, such as: ‘God is punishing me, does not like me and has abandoned me’. When these are present, outcomes tend to be negative with a greater prevalence of depression, anxiety and mortality (Pargament *et al.* 2001; Stratta *et al.* 2012).

Despite the extensive literature assessing correlations or associations between R/S and mental health, few studies have investigated the clinical applicability of this evidence through controlled clinical trials. Given this, some authors have proposed strategies to investigate whether the stimulation of religious/spiritual beliefs could result in better clinical outcomes (Koszycki *et al.* 2010; Ka'opua *et al.* 2011). It is believed that religious/spiritual interventions (RSI) have a role in changing an individual's thoughts, promoting greater acceptance of illness and social support and a deeper understanding of existence together with encouraging belief and faith, that could have an impact on patients’ outcomes (Djuric *et al.* 2009; Rosendahl *et al.* 2009).

Despite the growing number of studies, the approaches are still quite distinct and lack standardization. Some evaluate the increment of spirituality itself after the intervention (Richards *et al.* 2006), others evaluate quality of life (Moritz *et al.* 2006) and others the physical or mental health impact on patients (Huguelet *et al.* 2011). The difference between the protocols (frequency and duration) is also considerable, hindering comparisons between techniques.

Despite the theoretical evidence, at present, we found three meta-analyses comparing treatment involving R/S in the literature; however, these comprised heterogeneous treatment settings and selection criteria (McCullough, 1999; Smith *et al.* 2007; Oh & Kim, 2012).

In order to update and clarify the results found in the literature, the aim of the present study was to perform a systematic review following PRISMA (Preferred Reporting Items for Systematic reviews and Meta-Analyses) guidelines, selecting only randomized controlled trials, focusing on the impact of RSI on mental health outcomes, and to evaluate the methodological quality of these articles. Considering the heterogeneity of these studies, we aimed to perform a meta-analysis of studies capable of grouping through populations or clinical outcomes.

## Method

The present study is a systematic review and meta-analysis of randomized clinical trials involving RSI on mental health and it was conducted from January 2011 to June 2014.

### Eligibility criteria

Randomized clinical trials were eligible if they explored the effects of RSI on mental health outcomes without restrictions regarding the type of disease or population. RSI were considered to be ‘messages to health’ framed by themes of spiritual relevance. This ‘message’ could use spiritual or religious themes, such as taking care of the body God has provided (Anderson & Pullen, 2013), reflective discussions of moral and ethical values to accept the situation faced (Breitbart *et al.* 2010), or meditation (Bormann *et al.* 2008), among others.

Language was limited to English, Spanish and Portuguese; the date of publication, however, had no restriction. Due to the importance of an appropriate randomization process in clinical trials, we assumed as an exclusion criterion a randomization definition used by the CONSORT (Consolidated Standards of Reporting Trials) Group (Schulz *et al.* 2010), which comprises a checklist on how to report a trial. If the randomization procedure was not specified in articles, the authors were contacted.

### Search strategies

We screened the literature using seven databases: PubMed, Scopus, Web of Science, PsycINFO, The Cochrane Collaboration, Embase and SciELO. We decided to work with Boolean expressions, since these access relevant articles in a single expression (Pohl *et al.* 2010), as follows: ‘(spiritu* OR relig* OR faith OR holistic OR multifaith) AND (assistance OR intervention OR treatment OR therapy OR assessment OR group OR meditation) AND (clinical trial OR meta-analysis OR randomized controlled trial OR controlled clinical trial)’. A pilot search was run in June 2011 and updated in August 2013.

### Data abstraction

#### Phase 1

Two reviewers (Camila Casaletti Braghetta and J.P.B.G.) examined the title and abstract of studies to exclude those not comparing RSI with a control group, reviews, off-topic or in other languages and repeated versions in different databases.

#### Phase 2

Each included study went through an extensive review of the intervention and randomization processes. For articles without complete descriptions of adopted procedures, the authors were contacted by email for further information. Those who did not respond or provided insufficient information were excluded.

### Data items

Outcomes extracted from each included article were: (1) participants’ clinical diagnoses; (2) sample size; (3) intervention protocols (type, frequency, duration and follow-up); (4) scales and outcome measures; and (5) results of interventions. The outcomes explored in the meta-analysis were depressive and anxiety symptoms.

### Statistical analysis

To assess the risk of bias in the studies, we used the intraclass correlation coefficient which quantifies the percentage of data variability. The score of this coefficient ranges from 0 to 1.00; the closer to 1.00, the less variability exists between these measures. For this calculation, SPSS version 17.0 (SPSS Inc., USA) was used.

Regarding the meta-analysis, the Cochrane RevMan 5.2 program was employed. We chose to use random-effects models considering the possible heterogeneity in studies (Liberati *et al.* 2009), with a 95% confidence interval for each measure. In order to explore the variability of the results, we used a standard mean difference and an assessment of methodological quality in the articles.

Concerning the outcomes, in studies that used more than one control group, data were extracted and used as different analysis (e.g. Rosmarin *et al.* 2010.1 – control group *versus* RSI; Rosmarin *et al.* 2010.2 – other intervention or waiting list *versus* RSI).

With regards to heterogeneity, the Cochrane Collaboration classifies it into unimportant (0–40%), moderate (30–60%), substantial (50–90%) and considerable (75–100%) (Higgins & Green, 2011). In the present study, when heterogeneity was present, we explored possible explanations, by looking at subgroups, at type of intervention, type of control model and length of follow-up.

### Risk of bias in individual studies

Due to the nature of the RSI adopted, studies were not double-blind; therefore, we chose to evaluate the risk of bias of each study using the Cochrane Back Review Scale which contains 11 questions about methodology, providing a comprehensive assessment of important items of clinical trials (Berger & Alperson, 2009). Acceptable studies met at least six out of 11 validity criteria (Van Tulder *et al.* 2003). To check the validity of the analysis, three independent researchers (G.L., H.V., J.P.B.G.) rated the classification. Disagreements were discussed topic by topic and resolved by consensus.

## Results

### Selection of studies

**Fig. 1.**
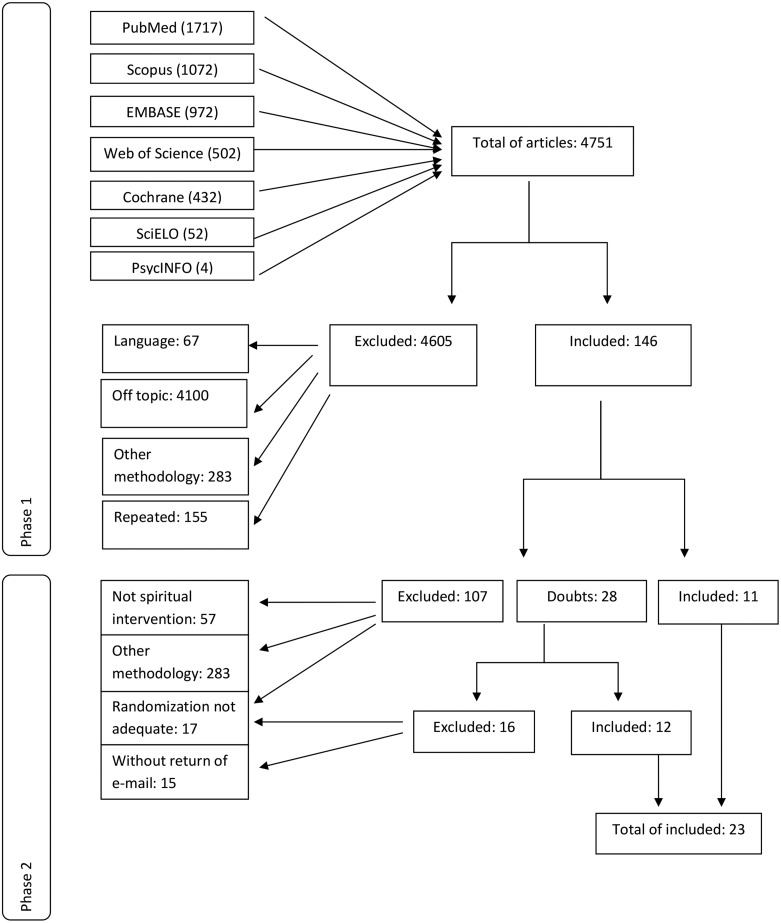
Flowchart of the selected studies following PRISMA (Preferred Reporting Items for Systematic reviews and Meta-Analyses) guidelines.

The survey of databases produced 4751 articles (see flowchart; Fig. 1). Phase 1 eliminated 4605 articles for not meeting the inclusion criteria: 4100 were out of theme and 283 had a different methodology, 155 were repeated citations and 67 were in other languages, resulting in 146 articles. In phase 2, 57 were excluded for not assessing spiritual interventions, 34 had a different methodology and 17 did not have adequate randomization. Doubts about randomization appeared in 28 articles. Authors were contacted by email, and despite 15 not responding, 12 studies were included of the 13 returned. These 12 were added to the 11 included in phase 2; a computed total of 23 papers.

### Characteristics of studies

Table 1 shows the general characteristics of the selected articles. The papers were published between 2005 and 2013, and 56.5% of these were from 2009 to 2013.

**Table 1.** Characteristics of studies

Author (year)	Participants	Sample size	Scale	Type of intervention	Number of sessions/duration of sessions, min	Follow-up, months	Results compared with control groups
Bay *et al.* (2008)	Cardiac presurgical	170	HADS, S S/R	Pastoral services	4/44	1 to 6	N/+
Bormann *et al.* (2006)	HIV+	93	Q-LES-Q, PSS, STAI, CES-D, S S/R	Meditation	5/90	1 to 6	N/N/+/+/+
Bormann *et al.* (2008)	Post-traumatic stress disorder	29	PTSD, Q-LES-Q, S S/R	Meditation	6/90	1 to 6	+/+/+
Bormann & Carrico (2009)	HIV+	93	STAI, S S/R	Meditation	5/90	1 to 6	+/+
Bowland *et al.* (2012)	Post-traumatic stress disorder	43	GDS, BAI, S S/R	Therapy	11/90	1 to 6	+/+/N
Breitbart *et al.* (2010)	Cancer	90	HADS, S S/R	Therapy	8/90	1 to 6	N/+
Breitbart *et al.* (2012)	Cancer	120	MQOL, HADS, S S/R	Therapy	7/60	1 to 6	+/N/N
Djuric *et al.* (2009)	Cancer survivors	24	Weight, 7D-PAR, CES-D, S S/R	Therapy	13/?	>6	+/N/+/+
Hosseini *et al.* (2013)	Cardiac presurgical	70	HAS	Pastoral services	5/45–60	<1	+
Huguelet *et al.* (2011)	Schizophrenia	84	PNSS, S S/R	Therapy	1+n/?	1 to 6	N/N
Kelly *et al.* (2011)	Drug users	774	Form90-D, S S/R	Therapy	12/60	>6	+/+
Koszycki *et al.* (2014)	Generalized anxiety disorder	23	HAS, PSWQ, BAI, BDI, S S/R	Therapy	12/50	1 to 6	+/+/N/N/+
Lloyd-Williams *et al.* (2013)	Cancer	100	BEDS, S S/R	Therapy	8/?	1 to 6	N/+
McCauley *et al.* (2011)	Chronic pain	100	Pain, MOOD, S S/R	Audiovisual sources	5/28	1 to 6	N/N/N
Miller *et al.* (2005)	Terminal patients	51	BDI, STAI, S S/R	Therapy	12/75	>6	N/N/N
Miller *et al.* (2008)	Drug users	64	Form90-D, BDI, STAI, S S/R	Audiovisual sources	12/?	<1	−/−/−/N
Moritz *et al.* (2006)	Emotional stress and depression	165	POMS, SF-36, S S/R	Audiovisual sources	8/90	1 to 6	+/+
Oman *et al.* (2006)	Healthy	58	PSS, MBI	Meditation	5/90	1 to 6	+/+
Oman *et al.* (2008)	Healthy	58	PSS, S S/R	Meditation	5/90	1 to 6	+/+
Rickhi *et al.* (2011)	Depression	84	HAM-D	Audiovisual sources	8/90	1 to 6	+
Rosmarin *et al.* (2010)	Generalized anxiety disorder	261	PSWQ, CES-D, S S/R	Audiovisual sources	14/30	>6	+/N/+
Wachholtz & Pargament (2005)	Healthy	84	STAI, Pain, S S/R	Meditation	14/20	1 to 6	+/N/+
Wachholtz & Pargament (2008)	Headache	83	ID-MS, Pain, STAI, CES-D, MSQFS, S S/R	Meditation	30/20	1 to 6	+/N/N/N/+

HADS, Hospital Anxiety and Depression Scale; S R/S, different scales of religiosity/spirituality; N, no difference between groups; +, positive effect; HIV+, human immunodeficiency virus-positive; Q-LES-Q, Quality of Life Enjoyment and Satisfaction Questionnaire; PSS, Perceived Stress Scale; STAI, Spielberger's State Anxiety Inventory; CES-D, Center for Epidemiological Studies Depression Scale; PTSD, Post-Traumatic Stress Disorder Scale; GDS, Geriatric Depression Scale; BAI, Beck Anxiety Inventory; MQOL, McGill Quality of Life Questionnaire; 7D-PAR, Seven-Day Physical Activity Recall; HAS, Hamilton Anxiety Scale; PNSS, Positive and Negative Syndrome Scale; Form90-D, alcohol questionnaire; PSWQ, Penn State Worry Questionnaire; BDI, Beck Depression Inventory; BEDS, Brief Edinburgh Depression Scale; MOOD, depressive symptoms; −, negative effect; POMS, Profile of Mood States; SF-36, Medical Outcomes Study 36; MBI, Maslach Burnout Inventory; HAM-D, Hamilton Depression Scale; ID-MS, ID Migraine Screener; MSQFS, Migraine Specific Quality of Life Scale.

Populations included sick and healthy people and represented a total sample of 2721 participants. Diagnoses included mental health disorders (26%), cancer (21.8%), chronic diseases (21.8%), substance use/abuse (8.7%) and cardiac conditions (8.7%), totalling 2521. The healthy population was composed of health professionals and other individuals (13%), including 200 people.

### Protocols of interventions

There were two main lines of approach in the selected papers: spiritual and religious. Spiritual approaches consisted of themes such as moral values, belief in a ‘high power’, coping and transcendence, and others in the form of therapeutic models, audiovisual resources and meditation. Religious approaches explored the beliefs and specific traditions of Catholics, Jews and Muslims, conducted in pastoral services and therapeutic models.

Many papers shared similar techniques grouped as follows: psychotherapy (nine studies); meditation (seven studies); audiovisual resources (five studies); and pastoral services (two studies), described below:

#### Psychotherapy

This method was predominant in the selected papers of which three followed a conventional therapeutic approach (e.g. cognitive–behavioural therapy), three an educational method and one evaluated both.

Three different types of control groups were found: therapeutic ones, educational approaches for disease and waiting list. The protocols varied from one to 12 sessions. Only two were held individually and the rest involved group discussions.

#### Meditation

In the seven papers about spiritual meditation, three associated an educational approach for the procedures. They compared the groups with traditional meditation, waiting list and informative videos about the disease concerned.

The facilitators of procedures were the authors who taught and answered questions about the exercises. Three requested that meditation be carried out as many times as possible during the day, while the rest counselled daily or weekly meditation sessions.

#### Audiovisual resources

In these interventions, authors constructed material as booklets, audios or videos for personal use, followed by questionnaires or discussion groups to debate absorbed ideas.

Two studies compared the intervention group with an informative group and waiting list, two only with waiting list and one with informative group.

Protocols of videos consisted of two with spiritual strategies for coping with the disease and one with Jewish beliefs. The booklets and audios were created and adapted mentioning spirituality focused on the disease treated, and also followed by discussion groups.

#### Pastoral services

Two studies in the form of chaplaincy were encountered, both in patients with preoperative cardiac programming. This approach was compared with a standard treatment approach in hospitals.

Chaplains followed guidelines for care, consisting of rituals (prayers, anointing, etc.) and spiritual support tailored to the medical needs of patients, such as hospitalization, postoperative complications, emotional and spiritual suffering. The sessions occurred pre- and postoperatively, with four visits in one study and at least five in the other. The time was not pre-set, but varied according to the needs of the patients in both studies.

### Outcomes and meta-analysis

Populations found were composed of patients and healthy individuals (Table 1). The mental health outcomes most assessed were depressive symptoms (found in 15 papers), anxiety (14 papers), post-trauma stress and stress levels (five papers), use/abuse of alcohol/drugs (two papers) and social function (one paper).

Among the usable results in the meta-analysis, three of depressive symptoms and two of anxiety were excluded because they did not present sufficient data for statistical tests (mean, standard deviation and/or standard error).

**Fig. 2.**
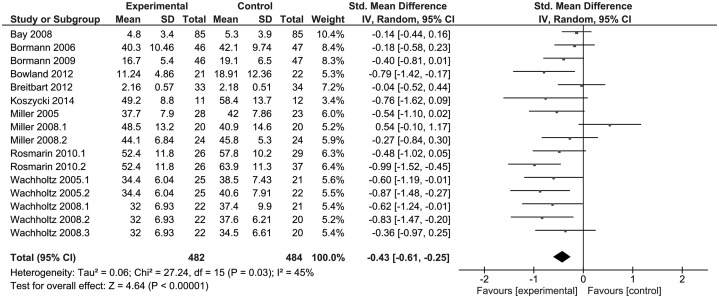
Forest plot of effect sizes for anxiety symptoms. SD, Standard deviation; IV, inverse variance; CI, confidence interval; df, degrees of freedom.

There was a statistical difference between the studies related to anxiety (*p* < 0.001) favouring RSI, presented in Fig. 2. We found evidence of high heterogeneity among the studies (*I*^2^ = 86%). After exploring the analysis, we identified a study with a low score in the Cochrane Scale; therefore it was treated as an outlier. The exclusion of these data was reflected in a resultant low heterogeneity (*I*^2^ = 45%).

**Fig. 3.**
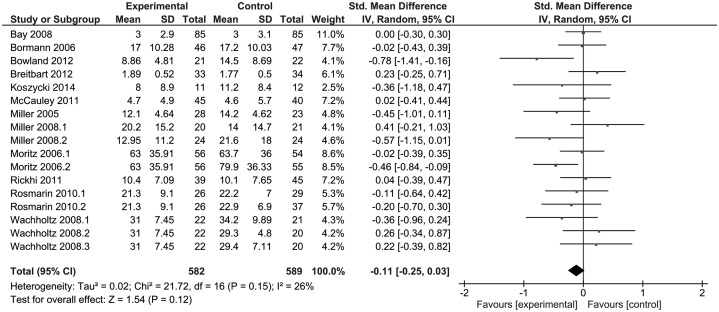
Forest plot of effect sizes for depressive symptoms. SD, Standard deviation; IV, inverse variance; CI, confidence interval; df, degrees of freedom.

Related to depressive symptoms there was no significant difference (*p* = 0.12), despite the tendency to favour RSI, as shown in Fig. 3. There was low evidence of heterogeneity (*I*^2^ = 26%).

In order to further explore the heterogeneity found in the studies related to anxiety (*I*^2^ = 45%) and the trend of benefit in depressive symptoms, we assessed three subgroups defined previously: types of intervention; follow-up; and types of control groups.

Related to anxiety, we found significant differences for meditation (*p* < 0.001 and *I*^2^ = 0%) and psychotherapy (*p* = 0.02 and *I*^2^ = 39%) (online Supplementary Fig. S1). There were also significant differences in three distinct moments of follow-up (online Supplementary Fig. S2), but the most impressive was assessments until 1 month post-interventions, without any heterogeneity between the studies (*I*^2^ = 0%). Regarding the control groups, studies that used any type of intervention showed a difference for R/S (*p* < 0.001 and *I*^2^ = 3%), when compared with waiting list groups (*p* = 0.19 and *I*^2^ = 73%) (online Supplementary Fig. S3).

Related to depressive symptoms, we found no differences between the types of intervention, although there was a trend with audiovisual resources and therapy (online Supplementary Fig. S4). We found, however, a difference in studies with follow-up from 1 to 6 months (*p* = 0.05 and *I*^2^ = 61%) (online Supplementary Fig. S5) and intervention control groups (*p* = 0.06 and *I*^2^ = 51%) (online Supplementary Fig. S6).

Concerning the results that were not usable in the meta-analyses due to the small numbers of each and/or outcomes measured, we describe the main results below:

(*a*) Healthy population: composed of four studies that explored mental health and satisfaction. One of them explored adolescents and found less anxiety, better humour and more spiritual experiences (Wachholtz & Pargament, 2005). The other three studies assessed health professionals and all showed lower levels of stress, emotional exhaustion, higher job satisfaction and even better quality of patient care (Oman *et al.* 2006, 2008; Huguelet *et al.* 2011).
(*b*) Use/abuse of alcohol/drugs: of two studies involving addicts that assessed the frequency and intensity of consumption, one showed a decrease that lasted after treatment (Kelly *et al.* 2011), and the other found a decrease in consumption only after 4 months, but increased rates of depression and anxiety in patients who received a RSI (Miller *et al.* 2008). Both showed an incorporation of spiritual practices and higher levels of faith.
(*c*) Post-trauma stress: two spiritual interventions showed promising results with significant reductions of post-trauma stress and a trend towards reductions in other psychological symptoms in men (Bormann *et al.* 2008) and women (Bowland *et al.* 2012).
(*d*) Schizophrenia: one study explored this disorder and noted increased social functioning, adherence to medical treatment and interest of patients in discussing spirituality with their psychiatrists (Huguelet *et al.* 2011).
(*e*) Migraine: participants who did a spiritual meditation demonstrated a reduction in the frequency of migraines and in levels of depressions and anxiety (Wachholtz & Pargament, 2008).

### Risk of bias in individual studies

We found an intraclass correlation coefficient of 0.832 (0.752–0.893) between examiners, showing the positive reliability of the assessment of bias risk. Table 2 discriminates the items assessed in the 23 final articles. It was noted that none reached the maximum score of 11 because these studies do not enable the use of ‘double-blind’ methods. The highest-scoring study at nine points was by McCauley *et al.* (2011). The ‘third-party blind’ method, which means that the examiner that has no idea of the patient's allocation, was present in four studies. There was uniformity of intensity, duration, frequency and follow-up (items H and J, respectively) in the protocols used.

**Table 2.** Description of the Cochrane Back Review Scale of methodological quality

Author (year)	A	B	C	D	E	F	G	H	I	J	K	Score
Bay *et al.* (2008)	+	+	+	−	−	−	+	+	+	+	−	7
Bormann *et al.* (2006)	+	+	+	−	−	−	+	+	+	+	+	8
Bormann *et al.* (2008)	+	+	+	−	−	?	+	+	+	+	?	7
Bormann & Carrico (2009)	+	+	+	−	−	−	+	+	+	+	+	8
Bowland *et al.* (2012)	+	+	+	−	−	+	−	+	+	+	+	8
Breitbart *et al.* (2010)	+	+	+	−	−	?	+	+	+	+	−	7
Breitbart *et al.* (2012)	+	+	?	−	−	?	+	+	+	+	−	6
Djuric *et al.* (2009)	+	+	+	−	−	?	+	+	+	+	−	7
Hosseini *et al.* (2013)	+	+	+	−	−	?	−	−	+	+	−	5
Huguelet *et al.* (2011)	+	+	+	−	−	−	+	+	+	+	−	7
Kelly *et al.* (2011)	+	+	+	−	−	+	+	+	−	+	+	8
Koszycki *et al.* (2014)	+	+	+	−	−	?	+	+	+	+	+	8
Lloyd-Williams *et al.* (2013)	+	+	−	−	−	?	?	+	−	+	?	4
McCauley *et al.* (2011)	+	+	+	−	−	+	+	+	+	+	+	9
Miller *et al.* (2005)	+	−	+	−	−	−	+	+	+	+	−	6
Miller *et al.* (2008)	+	−	+	−	−	−	−	+	+	+	+	6
Moritz *et al.* (2006)	+	+	+	−	−	?	−	+	+	+	+	7
Oman *et al.* (2006)	+	+	+	−	−	−	+	+	+	+	+	8
Oman *et al.* (2008)	+	+	+	−	−	−	+	+	+	+	+	8
Rickhi *et al.* (2011)	+	+	+	−	−	+	+	+	−	+	+	8
Rosmarin *et al.* (2010)	+	+	+	−	−	?	+	+	−	+	+	7
Wachholtz & Pargament (2005)	+	+	+	−	−	−	+	+	+	+	+	8
Wachholtz & Pargament (2008)	+	+	+	−	−	?	+	+	+	+	+	8

A, Randomization method; B, allocation concealed; C, similar baseline; D, patient blinded; E, provider blinded; F, assessor blinded; G, co-intervention avoided; H, acceptable compliance; I, acceptable drop-out; J, timing of outcome assessment similar; K, intention-to-treat analysis.

## Discussion

In order to respond to the need to develop the theme of RSI in terms of its clinical application and scientific impact, we performed a systematic review and meta-analysis. The results clearly showed that even RSI with different models, with distinct facilitators and populations tended to be associated with benefits, comparing results between both pre- and post-intervention groups, and control groups. The meta-analysis showed a significant reduction in anxiety levels and a trend towards improvement in depression. Despite the existence of other systematic reviews and meta-analyses of RSI, to our knowledge this is first time that such a report covers different scientific databases (a total of seven), and describes and discusses the methodology used in those selected studies in detail.

Despite the diversity of samples, the targeted goals converged into three basic groups: (*a*) evaluation of the impact of R/S on mental health; (*b*) comparison of the effect of R/S and conventional treatments described in the literature; and (*c*) verification of the acceptance and satisfaction of patients and facilitators in deployed research protocols. This is not an exclusive division because, according to the proposals of each study, some of them overlapped in their goals.

In our meta-analysis, statistical differences were found only in anxiety samples, with and without exploitation of heterogeneity. For depressive symptoms, the heterogeneity proved to be more suitable but there were no significant differences.

Previously, three meta-analyses compared conventional treatments with R/S. McCullough (1999) conducted a comparison of randomized studies of conventional therapeutic treatments and religious approaches in patients with predefined psychological symptoms. Of five studies selected, there was no difference to religious approach, suggesting that it should be done by patient's choice. Another meta-analysis conducted by Smith *et al.* (2007) examined 31 articles describing RSI in mental illness, showing a better clinical effect in patients when therapy included spiritual aspects. They included quasi-experimental studies and intervention without control group comparison. Nevertheless, neither review considered the methodological quality of the selected articles. A more recent study, by Oh & Kim (2012), followed PRISMA guidelines and included psychiatric diagnoses in addition to other health problems. Statistical differences were demonstrated for depression and anxiety in spiritual complementary treatments, with a sample of high heterogeneity (*I*^2^ = 94% in both cases). Their selection included studies of intercessory prayer and distance healing, besides clinical trials without randomization.

All protocols involving RSI had positive or neutral results – after comparisons with the control groups or between pre- and post-intervention in the same group – with one exception, in which patients had negative outcomes when compared with the control group (Miller *et al.* 2008). Each of these studies had its particularities, but, in general, they demonstrated reliability of using R/S as a complementary treatment (Tuck & Thinganjana, 2007; Stein *et al.* 2013).

Currently, several complementary treatments have been used to treat chronic diseases, minimizing symptoms and improving quality of life. We can cite psychotherapy, physical exercises, acupuncture and yoga among others (McCullough, 1999; Allen *et al.* 2006; Cramer *et al.* 2013; Underwood *et al.* 2013).

In our subgroup analysis, we searched for different impacts of RSI by dividing them into types of intervention, follow-up and types of control groups.

Regarding the types of intervention, we found evidence of efficacy in meditation and psychotherapy for anxiety symptoms. Although we found no difference between the types of intervention for depressive symptoms, the meta-analysis graphics showed a trend towards better results in audiovisual and therapeutic approaches.

We found in the literature two meta-analyses on different meditation techniques for both symptoms. Both studies showed positive effects, with the strongest evidence for anxiety (Abbott *et al.* 2014; Chan & Larson, 2015). However, none of the authors mentioned religious/spiritual meditation focus, which makes difficult the comparison with our study. There is little evidence to understand the role of spiritual meditations on mental health symptoms.

Hook *et al.* (2010) compiled a review about religious and spiritual therapies on mental health problems. They argued that several types of therapies were able to help different psychological problems, such as therapy based on religiosity can be more effective than other secular therapies and even some drug treatments. They showed strong evidence for anxiety for different religious therapies and Christian meditation, and all of them showed benefits between 1 and 3 months of follow-up, in accordance with our meta-analysis. Although only two types of therapies met their criteria for efficacy, Hook *et al.* (2010) discussed that this was due to insufficient evidence and not because these therapies do not work. Recently, Nyer *et al.* (2013) compiled a review on the role of complementary treatments in depression, showing that therapies based on R/S and music therapy showed improved outcomes in patients, but still with little evidence.

Some studies about self-help interventions on mental health (audiovisuals) have shown promising results, especially for patients with depression, a population that seems to benefit most from these interventions (Reins *et al.* 2013; Fuhr *et al.* 2014; Matcham *et al.* 2014). A recent meta-analysis evaluated, among other subgroups, the impact of this type of intervention in patients with depression using different lengths of follow-up, and found statistical difference between 1 and 3 months post-intervention (Matcham *et al.* 2014). In our study, we found statistically significant differences in interventions for depressive symptoms between 1 and 6 months of follow-up, although there was no distinction between the types of intervention. Sarris *et al.* (2014) mentioned in their review that different approaches can and should be used in patients with depression encouraging changes in lifestyle and this can take a while to adjust.

Following a methodological direction, we reinforce the importance of assessing the risks of bias in studies. According to PRISMA, there is a need to investigate this carefully through scales that examine the research item by item (Liberati *et al.* 2009). Regarding clinical trials, they cite the importance of allocation concealment for the randomization procedure, since its inadequacy may affect the results. Studies with similar methodologies, but discrepancies in quality, may have biased results (Liberati *et al.* 2009). This research considered adequate randomization as an inclusion criterion; among all articles, only three possessed a score below the cut.

The Cochrane Scale assesses, among other things, the randomization process and whether the allocation sequence was performed by an independent person who has no influence on the eligibility of patients, since these strategies improve the quality of research (Jadad, 1998; Liberati *et al.* 2009). The success of randomization depends on two interrelated aspects: generating an appropriate sequence of unpredictable allocation and concealment of the sequence until assignment occurs (Altman *et al.* 2001). The choice of the randomization procedure and its description in scientific papers therefore impose differences in the structure of the research.

There are several ways to prepare randomization allowing options for the more convenient and less expensive form for development studies. It is noteworthy that if authors have not described this procedure, it does not necessarily imply that they have not done it. However, we should remember that adequate description of the randomization procedure is essential in clinical research.

Another point to observe is the limitation for the item ‘double-blind’, since exploration in RSI happens with the knowledge and active participation of the patient, which makes it impossible to ‘blind’ the patient and the facilitator. Despite the importance of this item to minimize bias in clinical trials, modified guidelines of CONSORT for non-pharmacological approaches do not invalidate research without it, but suggest it to be justified in relation to the limitations of the procedure (Boutron *et al.* 2008). There are several examples of studies that have this type of restriction, such as research in psychotherapy, where at least applicators are aware of the procedure performed (Belotto-Silva *et al.* 2012; Devereaux *et al.* 2002).

A way to minimize difficulties in studies faced with problems with double-blinding is to use a ‘third-party blind’, an assessment that is not aware of patient's allocation, so the evaluation of patients can be conducted with impartiality. Only 17.9% of studies used ‘third-party blinding’, showing that this strategy still needs to be considered and explored in future research.

We also observed that, regardless of the evaluated protocols and studied populations, there was a general concern about the intensity, duration, frequency and follow-up results of interventions. In studies of high quality, the authors chose to present protocols that had similarities between the RSI and control groups, detailing all the deployed processes. Our meta-analysis of subgroups presented a statistical difference in interventions that used some procedure for comparison groups *versus* waiting list groups, but it also can be explained by the high heterogeneity between the studies.

The analysis of quality has revealed important aspects to be considered when producing clinical research on RSI. According to the natural difficulties already addressed in this line of research, other relevant aspects for minimizing biases are important and easily applicable. Attempts at improving the methodological issues of R/S studies may make a difference to finding more credible and reliable answers to questions regarding this topic.

### Limitations

This research has some limitations regarding the review and meta-analysis. Concerning the systematic review: (*a*) the definition of RSI adopted in the survey may have limited the access to some clinical trials; (*b*) the option of limiting the languages may have excluded other articles; and (*c*) although the assessment included seven databases, it is possible that some studies indexed in other databases have not been included, as well as articles published only in books or proceedings of congresses.

### Future directions for research

The need for more studies is clear, especially to understand the effects and mechanisms of action of RSI to health. Despite there being few studies that show clinical worsening with negative religiosity (Pargament *et al.* 2001; Stratta *et al.* 2012), one should consider these data to explore the pathways of R/S that can bring the benefits shown by many other studies. Adherence to the CONSORT guidelines in respect to clinical trial steps and the consequent production of quality research may help to reveal the benefits of these interventions. The use of appropriate randomization protocols, employing a ‘third-party blind’ method and considering ‘intent to treat’ are steps that can be included in these studies that can make a difference when minimizing biases.

An interesting point for future research would be to compare RSI employing different scales that measure spirituality, religiosity and daily spiritual practices among other measures already validated, in order to identify the possible mechanisms of action of this proposal.

## Conclusion

Clinical trials assessing the effects of RSI showed additional benefits compared with control groups, including reduction of clinical symptoms (especially levels of anxiety). The diversity of protocols and outcomes associated with the lack of standardization of interventions points to the need for more studies evaluating the use of spirituality as a complementary health treatment.

## Supplementary material

For supplementary material accompanying this paper visit http://dx.doi.org/10.1017/S0033291715001166. click here to view supplementary material
