# Supplementary material for: Religious and spiritual interventions in mental health care: a systematic review and meta-analysis of randomized controlled clinical trials
Source: Psychol Med. 2015 Jul 23;45(14):2937–49. doi: 10.1017/S0033291715001166 (PMC4595860; doi:10.1017/S0033291715001166)
Supplement: Supplementary file 1 [file S0033291715001166sup001.doc]

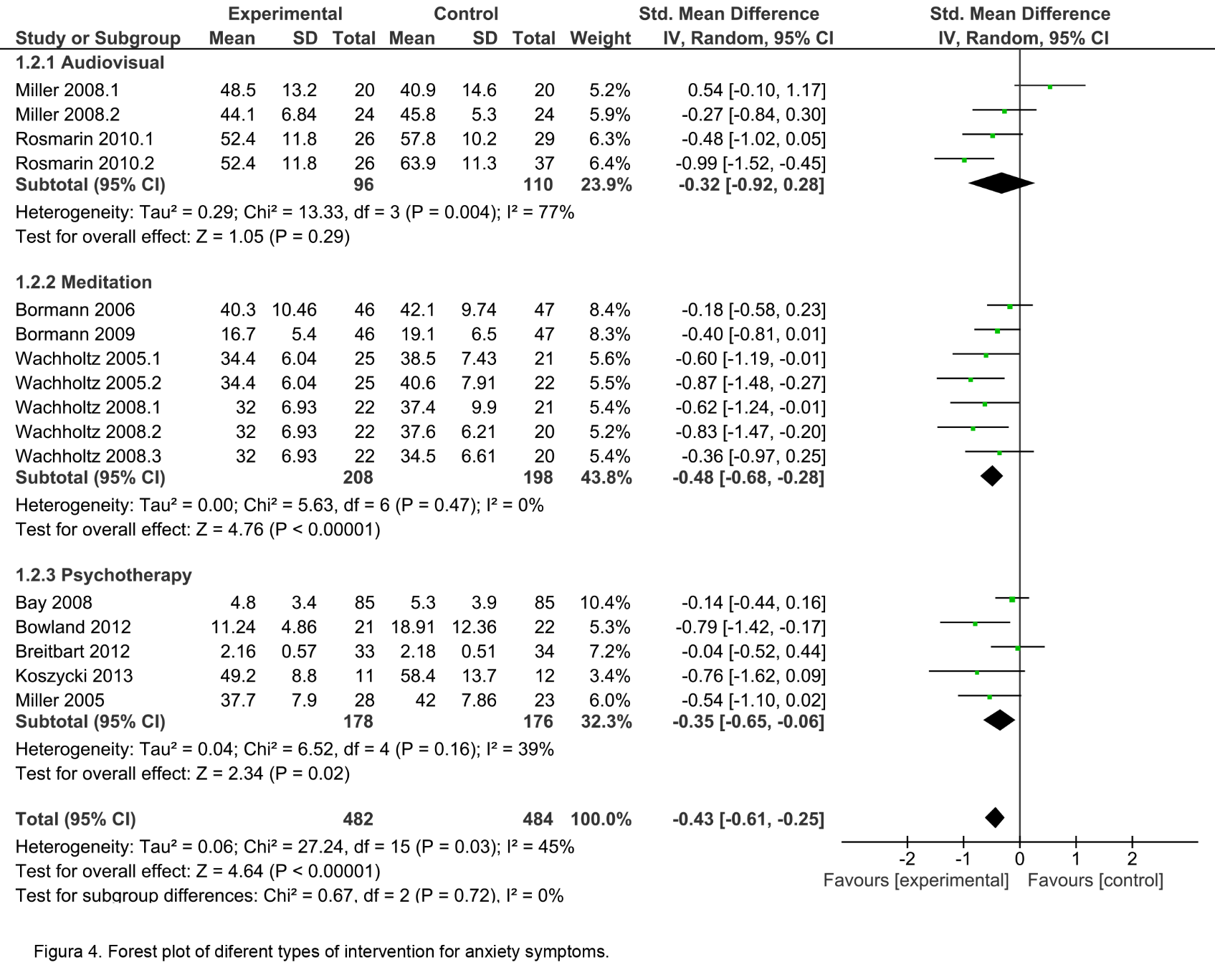


**Fig. S1.** Forest plot of different types of intervention for anxiety symptoms. See reference Koszycki *et al.* (2014) for the study Koszycki 2013.


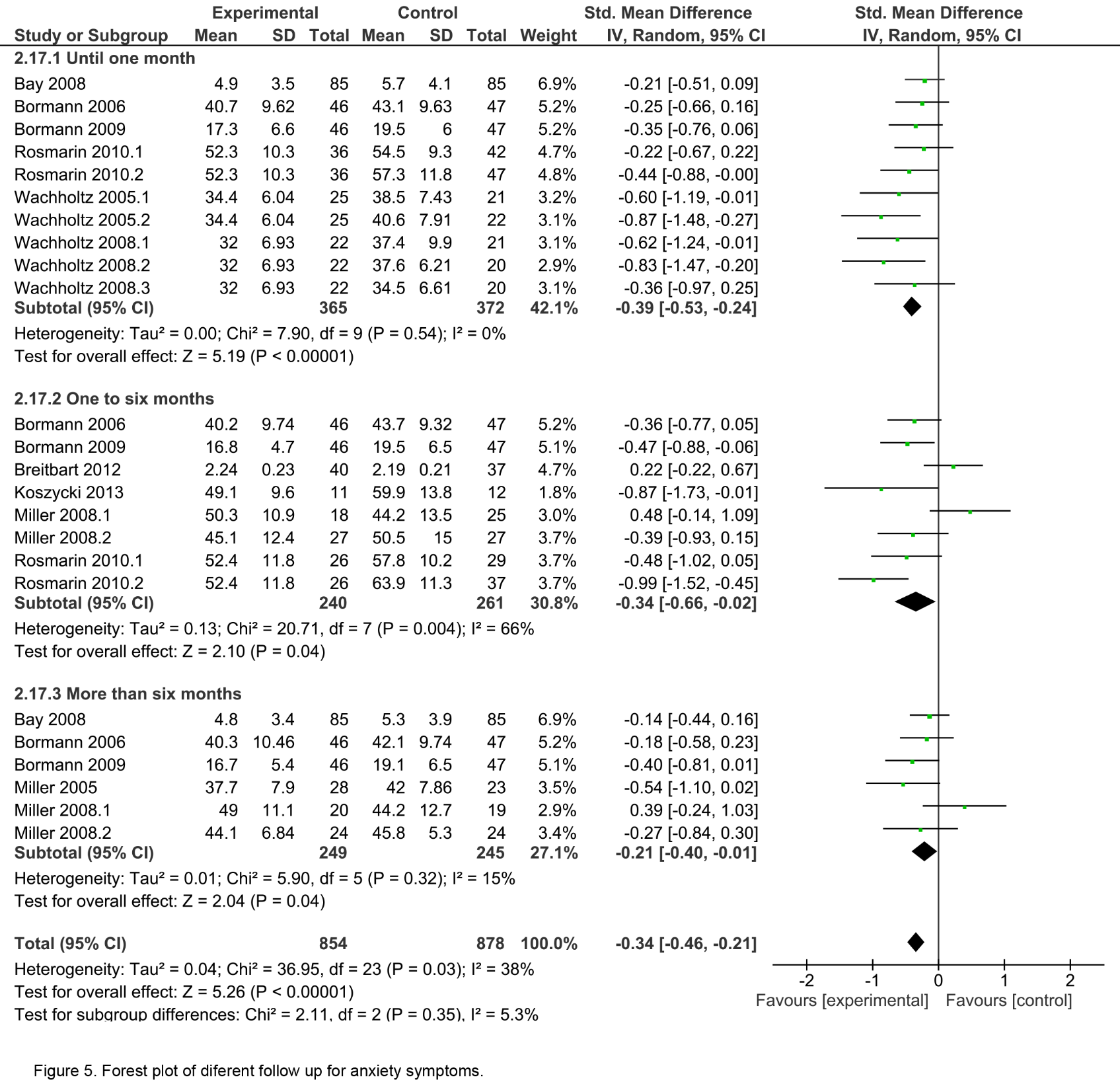


**Fig. S2.** Forest plot of different follow-up for anxiety symptoms. See reference Koszycki *et al.* (2014) for the study Koszycki 2013.


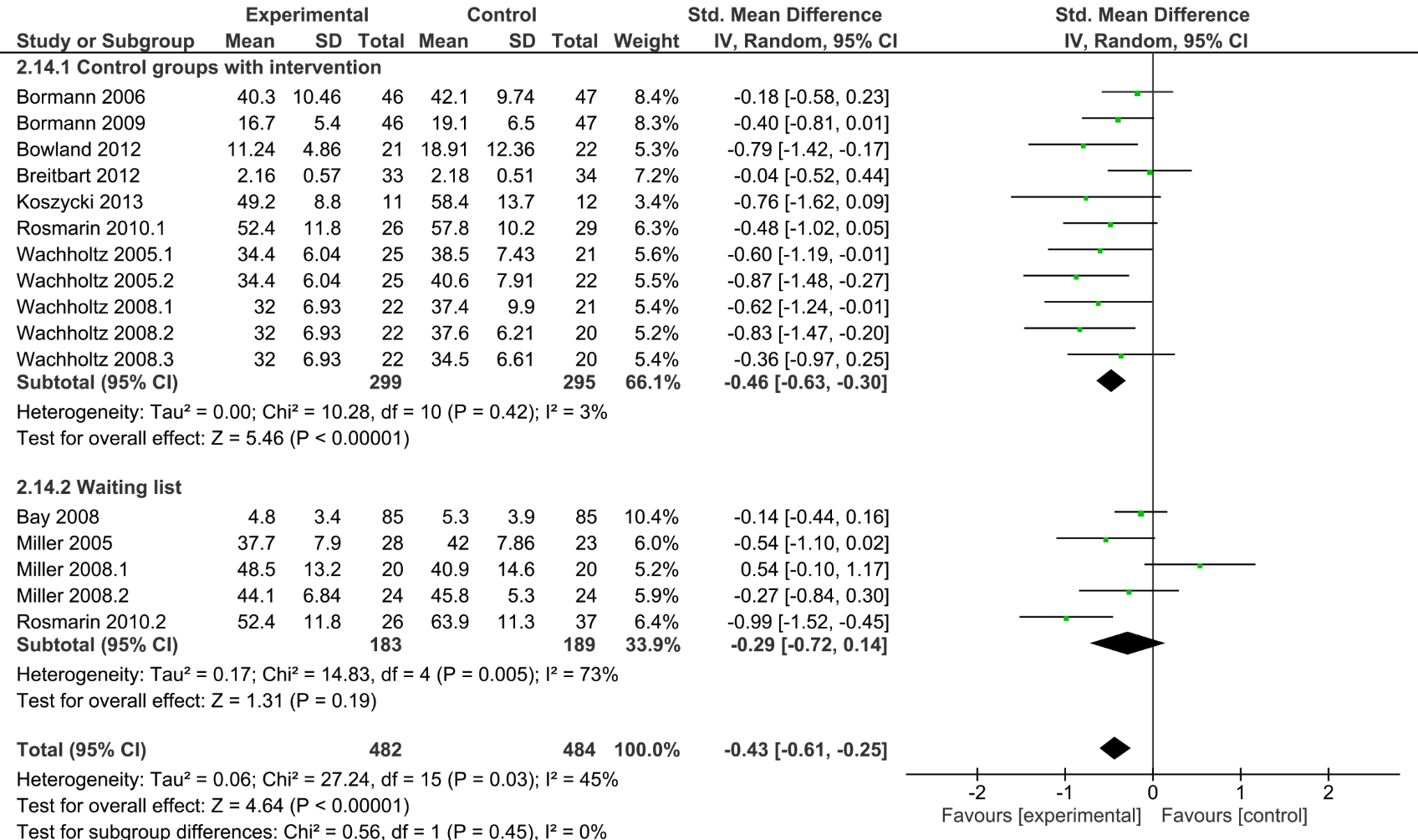


**Fig. S3.** Forest plot of different control groups for anxiety symptoms. See reference Koszycki *et al.* (2014) for the study Koszycki 2013.


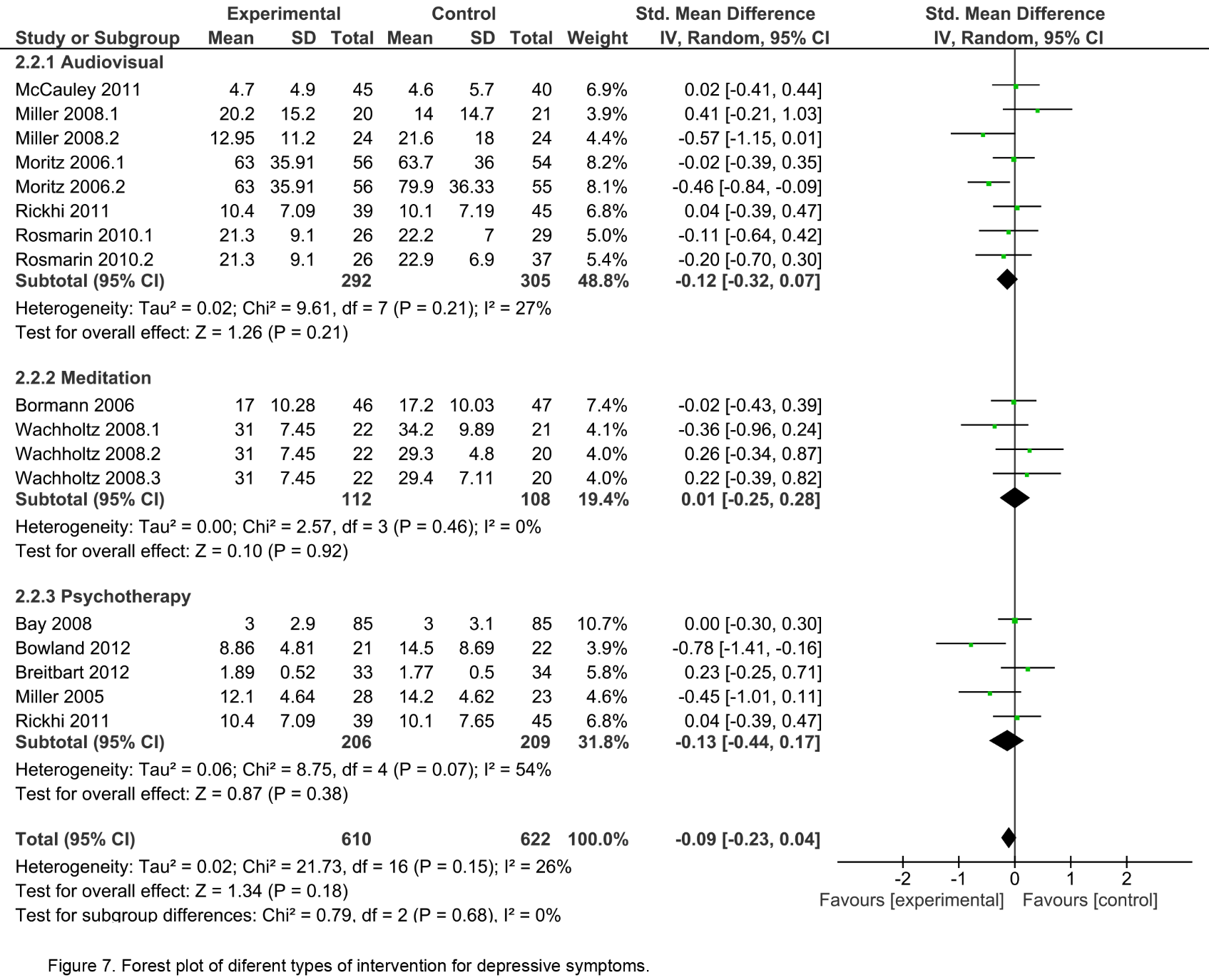


**Fig. S4.** Forest plot of different types of intervention for depressive symptoms.


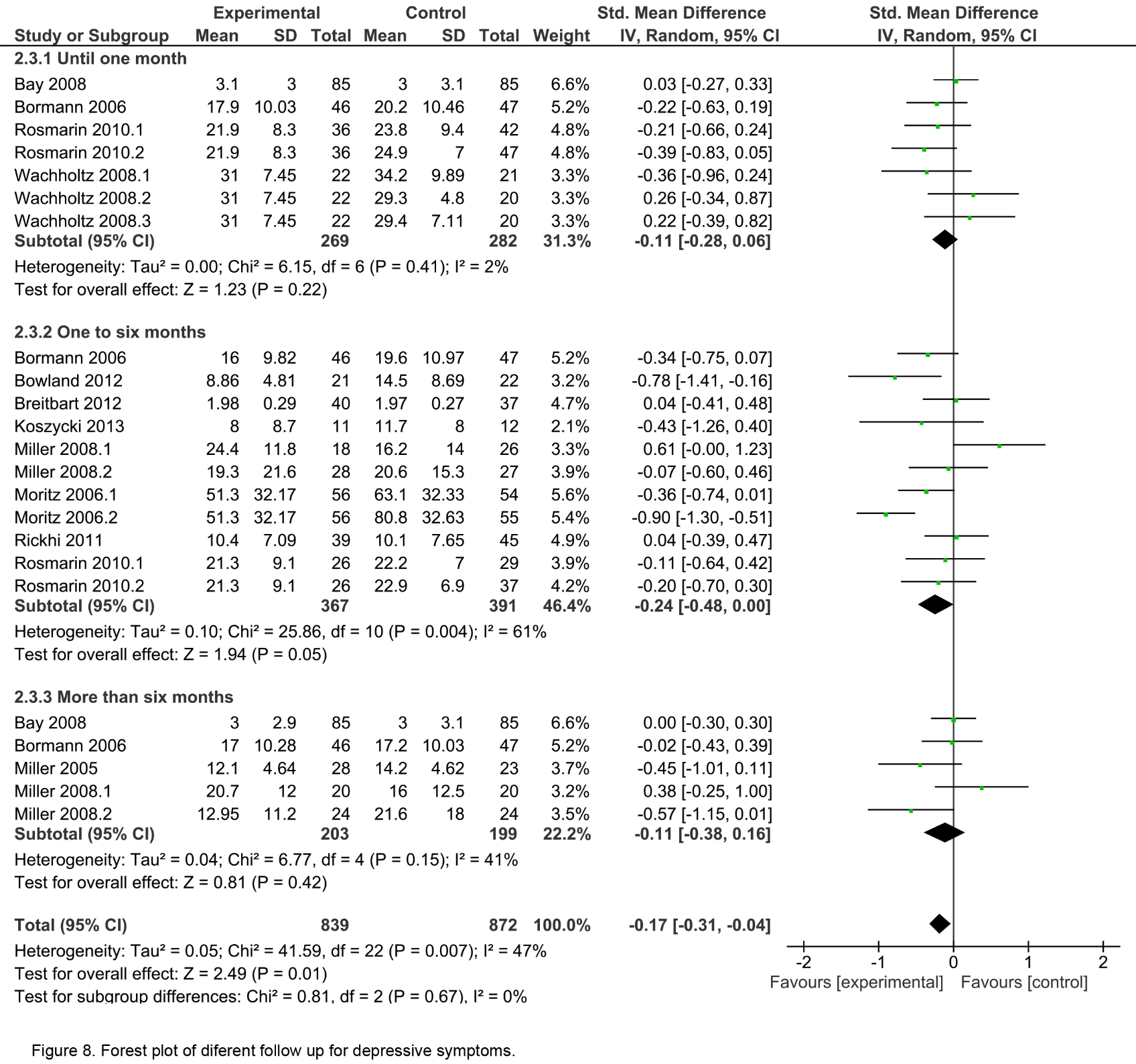


**Fig. S5.** Forest plot of different follow-up for depressive symptoms. See reference Koszycki *et al.* (2014) for the study Koszycki 2013.


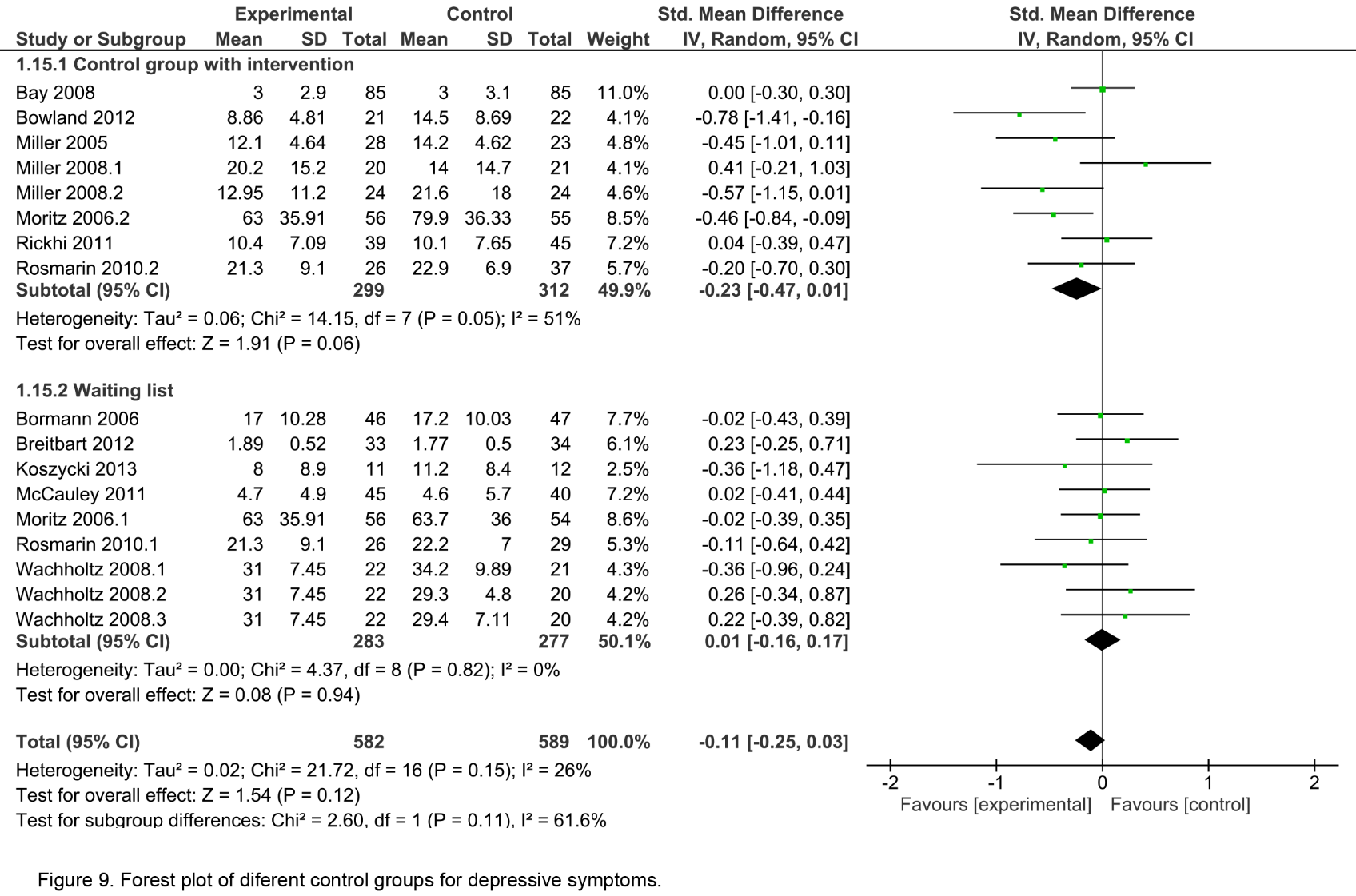


**Fig. S6.** Forest plot of different control groups for depressive symptoms. See reference Koszycki *et al.* (2014) for the study Koszycki 2013.
